# The association between metabolic syndrome and erosive esophagitis: A systematic review and meta-analysis

**DOI:** 10.17179/excli2021-4282

**Published:** 2021-11-08

**Authors:** Milad Azami, Majid Salamati, Reza Ranjbar, Amirhossein Sahebkar

**Affiliations:** 1 Faculty of Medicine, Ilam University of Medical Sciences, Ilam, Iran; 2 Molecular Biology Research Center, Systems Biology and Poisonings Institute, Baqiyatallah University of Medical Sciences, Tehran, Iran; 3 Applied Biomedical Research Center, Mashhad University of Medical Sciences, Mashhad, Iran; 4 Biotechnology Research Center, Pharmaceutical Technology Institute, Mashhad University of Medical Sciences, Mashhad, Iran; 5 School of Medicine, The University of Western Australia, Perth, Australia; 6 School of Pharmacy, Mashhad University of Medical Sciences, Mashhad, Iran

**Keywords:** metabolic syndrome, erosive esophagitis, meta-analysis

## Abstract

Although several studies have shown that each of the metabolic syndrome (MetS) components can be a risk factor for erosive esophagitis (EE), the association between MetS and EE is still a challenging subject, as studies about this association have shown inconsistent results. Therefore, this study was conducted to evaluate the association between MetS and EE. In this study, we followed the MOOSE protocol and the PRISMA guidelines for reporting the results. Web of Science (ISI), Cochrane Library (Cochrane Database of Systematic Reviews - CDSR), EMBASE, Scopus, Science Direct, PubMed/Medline, EBSCO, CINAHL, and Google Scholar search engine were searched for articles published until January 2021. Heterogeneity between studies was estimated by I2 index and Q test. All analyses were performed using Comprehensive Meta-Analysis Software. Finally, 12 studies entered the meta-analysis process after qualitative assessment. MetS was significantly associated with increased risk of EE (OR=1.488 [95 % CI: 1.352-1.638], P<0.001; Heterogeneity: I2= 55.57, P<0.001) in 12 studies with a sample size of 45285 (12825 cases and 29377 controls). In subgroup analysis based on types of studies (P=0.832), MetS diagnostic criteria (P=0.083) and quality of studies (P=0.612), no significant association was found. Sensitivity analysis showed that the overall estimation of effect size is still robust after omission of individual studies from the meta-analysis. Publication bias based on the Begg's test (P=0.945) and Egger's test (P=0.753) were not significant. MetS increases the risk of EE compared to control groups. Future studies should examine if MetS treatment reduces the risk of EE.

## Introduction

Gastroesophageal reflux disease (GERD), also known as acid reflux, is a long-term condition in which stomach contents rise into the esophagus, resulting in either symptoms or complications. This uncommon flow of gastroesophageal contents, which is caused by chronic exposure to esophageal epithelium with esophagitis, may cause esophageal mucosal damage, bleeding, or ulcers (Chiba et al., 2012[8]; Kahrilas, 2003[30]; Vakil et al., 2006[51]). GERD is the most common upper gastrointestinal disease in Western countries, while 10 to 20 % of the population having weekly symptoms (Cappell, 2005[7]; Hunt et al., 2007[28]; Miwa, 2006[35]). Its prevalence has increased in the Far East (Japan) and other regions of Asia (Fock et al., 2008[16]). Based on the findings of esophagogastroduodenoscopy, GERD is classified into three categories: non-erosive esophagitis (Non-EE), erosive esophagitis (EE), and Barrett’s esophagus (BE) (Ierardi et al., 2010[29]). EE refers to tissue changes in the esophageal mucosa in upper endoscopy, which has become a major health problem in Western countries, and epidemiological studies indicate that its incidence is increasing (Goh, 2011[18]). 

Metabolic syndrome (MetS) is a complex disorder that includes central obesity, hyperglycemia, hypertension, high-density lipoprotein cholesterol (HDL-C) and hypertriglyceridemia. In addition to being associated with cardiovascular disease and diabetes, MetS and its components are also associated with various gastrointestinal diseases, abnormal liver function, and polycystic ovary syndrome (Cooper-DeHoff and Pepine, 2007[11]; Hsieh et al., 2009[24]; Otaghi et al., 2019[40]). The disease has affected one-fifth of the population in developed countries and its incidence increases with age. The prevalence of MetS is about 24 % in the United States, 12 % in Europe and 10 to 40 % in most Asian countries (Ryan et al., 2008[44]; Tan et al., 2004[49]).

Recent literature has hypothesized the relationship between MetS and GERD (Wu et al., 2011[53]). Most studies that examine the association between obesity and GERD have shown that obesity can significantly increase the risk of GERD and EE symptoms (Bechade et al., 2009[4]; El-Serag, 2008[13][14]; Hampel et al., 2005[20]; Piretta et al., 2007[42]). Although several studies have shown that each of the MetS components can be a risk factor for EE, the association between MetS and EE is still a challenging subject, as studies about this association have shown inconsistent results (Chua et al., 2009[9]; Chung et al., 2008[10]; Hsieh et al., 2019[25]; Hsu et al., 2011[26]; Hung et al., 2016[27]; Lee et al., 2017[33]; Loke et al., 2013[34]; Niigaki et al., 2013[39]; Park et al., 2008[41]; Tai et al., 2010[48]; Wu et al., 2011[53]). 

Meta-analysis is a statistical method for combining data of several studies with similar goals. When the effect size is consistent between two studies, meta-analysis can be used to identify this common effect. When the effect is different between two studies, meta-analysis may be used to identify the cause of the inconsistency. Finally, meta-analysis results may include a more accurate estimate of the impact of treatment or risk factors for the disease or other outcomes by combining different studies (Azami et al., 2019[2]; Badfar et al., 2018[3]). This study was conducted to evaluate the association between MetS and EE.

## Methods

### Study protocol

In this study, we followed the Meta-analyses Of Observational Studies in Epidemiology (MOOSE) (Stroup et al., 2000[47]) protocol and the Preferred Reporting Items for Systematic Reviews and Meta-analysis (PRISMA) (Moher et al., 2015[37]) guidelines for reporting the results. The search, study selection, data extraction and qualitative assesment of the selected studies were carried out by at least two reviewers (M.A. and M.S.) and the disagreements were resolved through concordance and group discussion. The study protocol was not published before this study.

### Search strategy

Eight online databases were searched for articles published until January 2021: Web of Science (ISI), Cochrane Library (Cochrane Database of Systematic Reviews - CDSR), EMBASE, Scopus, Science Direct, PubMed/Medline, EBSCO, CINAHL, and Google Scholar search engine.

The search was done using the following MeSH keywords: "Metabolic Syndrome"[Mesh], "Gastroesophageal Reflux"[Mesh], "Esophagus"[Mesh], "Esophagitis"[Mesh]. Combined search in PubMed was done as follows: ((("Esophagus"[Mesh]) OR "Gastroesophageal Reflux"[Mesh]) OR "Esophagitis"[Mesh]) AND "Metabolic Syndrome"[Mesh]. Potential articles were also obtained by manual search in the reference list from a review article published in 2016 (Mohammadi et al., 2016[36]) and manual search of the selected articles.

### Inclusion and exclusion criteria

Inclusion criteria were: published articles, with abstract, English language studies that examined the relationship between MS and EE. Exclusion criteria were: duplicate studies, studies that did not present a correct definition of MetS, studies that did not differentiate the effect of EE from GERD, studies not relevant to our subject, congress, letters to the editor, studies lacking qualitative data and theses.

### Article selection

Titles and abstracts of all identified reports were reviewed. The full text of articles was evaluated according to the inclusion and exclusion criteria. Eventually, the disagreements were discussed and resolved in the presence of all authors.

### Data extraction

The following data were extracted from each study: first author, year of publication, type of study, country/continent, setting, mean age and standard deviation, population size of studies (total, case, control, male and female in case group and control group), the number of patients with MetS in case group and control group, odds ratio (OR), or relative risk (RR), and 95 % confidence interval (CI), MetS diagnosis criteria, EE diagnostic criteria and the qualitative assessment score of the studies.

### Quality assessment

For this purpose, the Newcastle-Ottawa Scale (NOS) was used to evaluate the quality of nonrandomized studies (case-control and cohort) (Wells et al., 2011[52]). In addition, adapted Newcastle-Ottawa Scale was used for the assessment of cross-sectional studies. The maximum score was 9. Three categories were defined for the quality of studies: low quality (score less than 5), average quality (score 6-7) and high score (score 8-9).

### Statistical analysis

All analyses were performed using Comprehensive Meta-Analysis Software (CMA). We used ORs index and 95 % confidence interval to combine the initial studies and finally reported the results as OR and 95 % CI. In studies that did not report ORs and 95 % confidence intervals, we found them based on the total sample size of each group as well as the number of MetS positive patients in case (EE) and control (Non-EE) groups. We performed the meta-analysis using random and fixed effects model. Heterogeneity between studies was estimated by I2 index and Q test (Ades et al., 2005[1]; Higgins, 2008[22]). To find the cause of high heterogeneity between the studies, we performed meta-regression and subgroup analysis. Sensitivity analysis was performed by sequential omission of each individual study to test the stability of the result of meta-analysis. Publication bias was assessed using Egger's test and Begg's test (Begg and Mazumdar, 1994[5]; Egger et al., 1997[12]). P values below 0.05 were considered statistically significant.

## Results

### Search results and characteristics of studies

**Figure 1:**
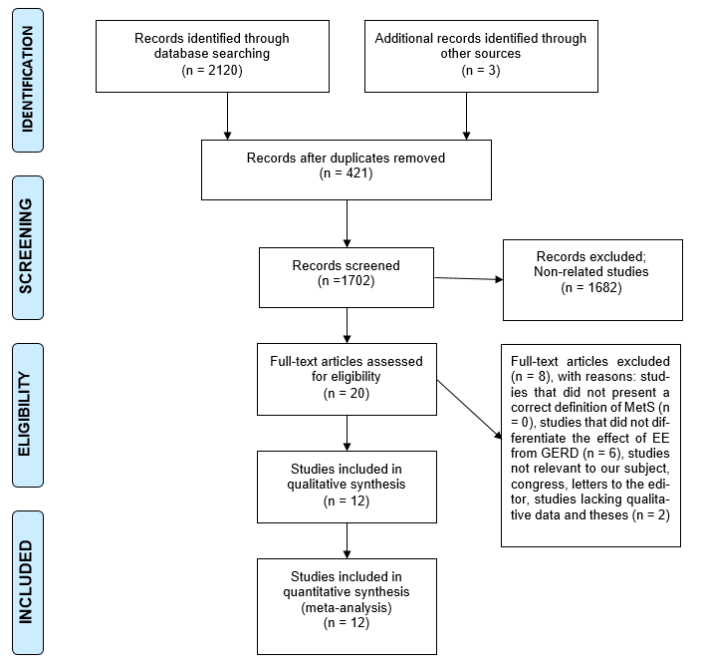
The studies selection process for meta-analysis

Figure 1(Fig. 1) shows the flowchart of the screening and selection of studies. The electronic search yielded 2123 entries and manual search identified seven more studies. The articles were reviewed based on the titles and abstracts, resulting in the exclusion of 421 duplicate and 1682 unrelated articles. Eight studies were excluded after full-text review because they did not meet the inclusion criteria. Finally, 12 studies entered the meta-analysis process after qualitative assessment (the study by Hung et al. (2016[27]) was considered as two studies, since it reported the data in two different populations) (Figure 1(Fig. 1)). All studies were conducted in Asia and had appropriate quality to be included in meta-analysis (Table 1(Tab. 1); References in Table 1: Chua et al., 2009[9]; Chung et al., 2008[10]; Hsieh et al., 2019[25]; Hsu et al., 2011[26]; Hung et al., 2016[27]; Lee et al., 2017;[33] Loke et al., 2013[34]; Niigaki et al., 2013[39]; Park et al., 2008[41]; Tai et al., 2010[48]; Wu et al., 2011[53]).

### MetS and increased risk of EE

**Figure 2:**
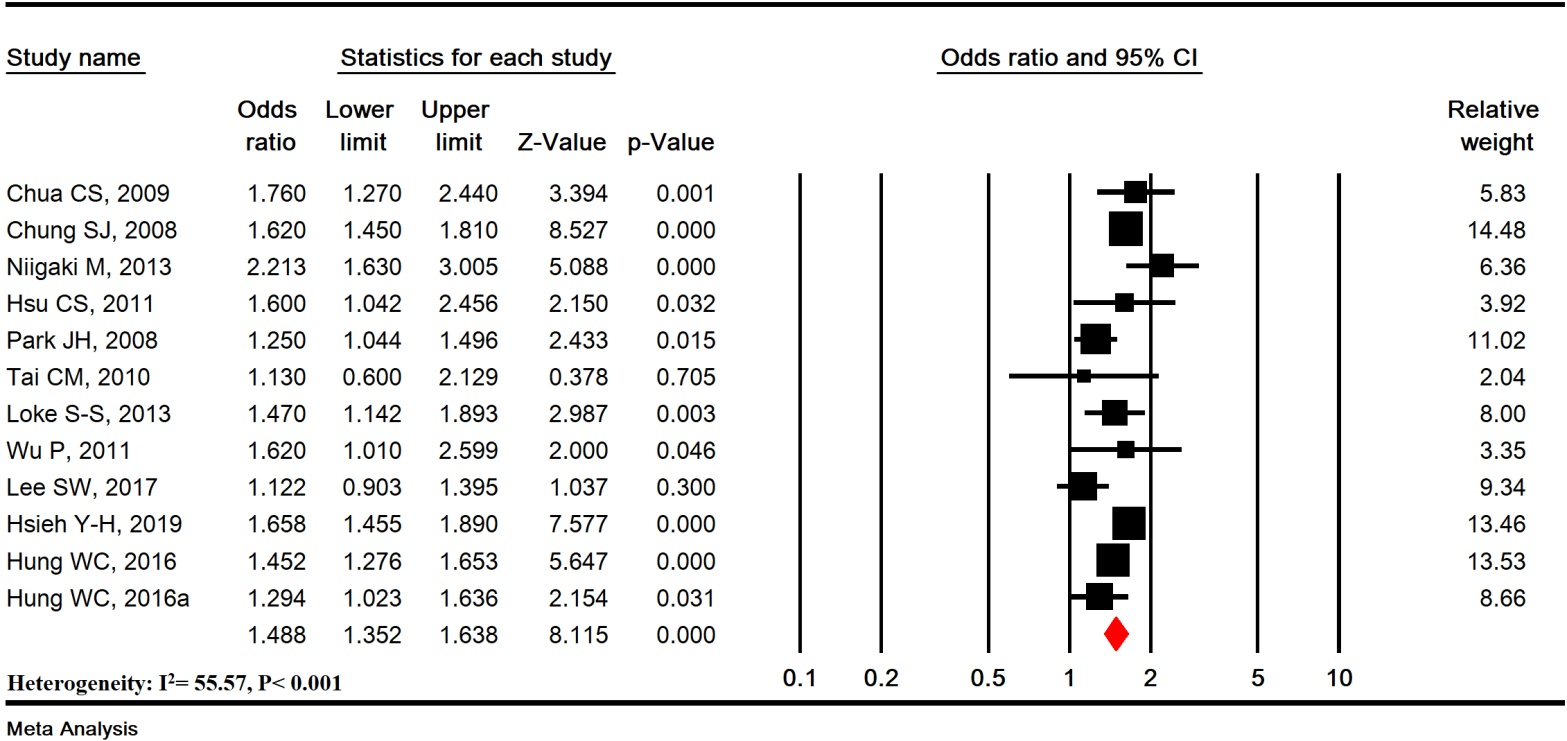
The association between metabolic syndrome and increased risk of erosive esophagitis

MetS was significantly associated with increased risk of EE (OR=1.488 [95 % CI: 1.352-1.638], P<0.001; Heterogeneity: I2= 55.57 %, P<0.001) in 12 studies with a sample size of 45285 (12825 cases and 29377 controls) (Figure 2(Fig. 2); References in Figure 2: Chua et al., 2009[9]; Chung et al., 2008[10]; Hsieh et al., 2019[25]; Hsu et al., 2011[26]; Hung et al., 2016[27]; Lee et al., 2017[33]; Loke et al., 2013[34]; Niigaki et al., 2013[39]; Park et al., 2008[41]; Tai et al., 2010[48]; Wu et al., 2011[53]).

### Sensitivity analysis and cumulative analysis

**Figure 3:**
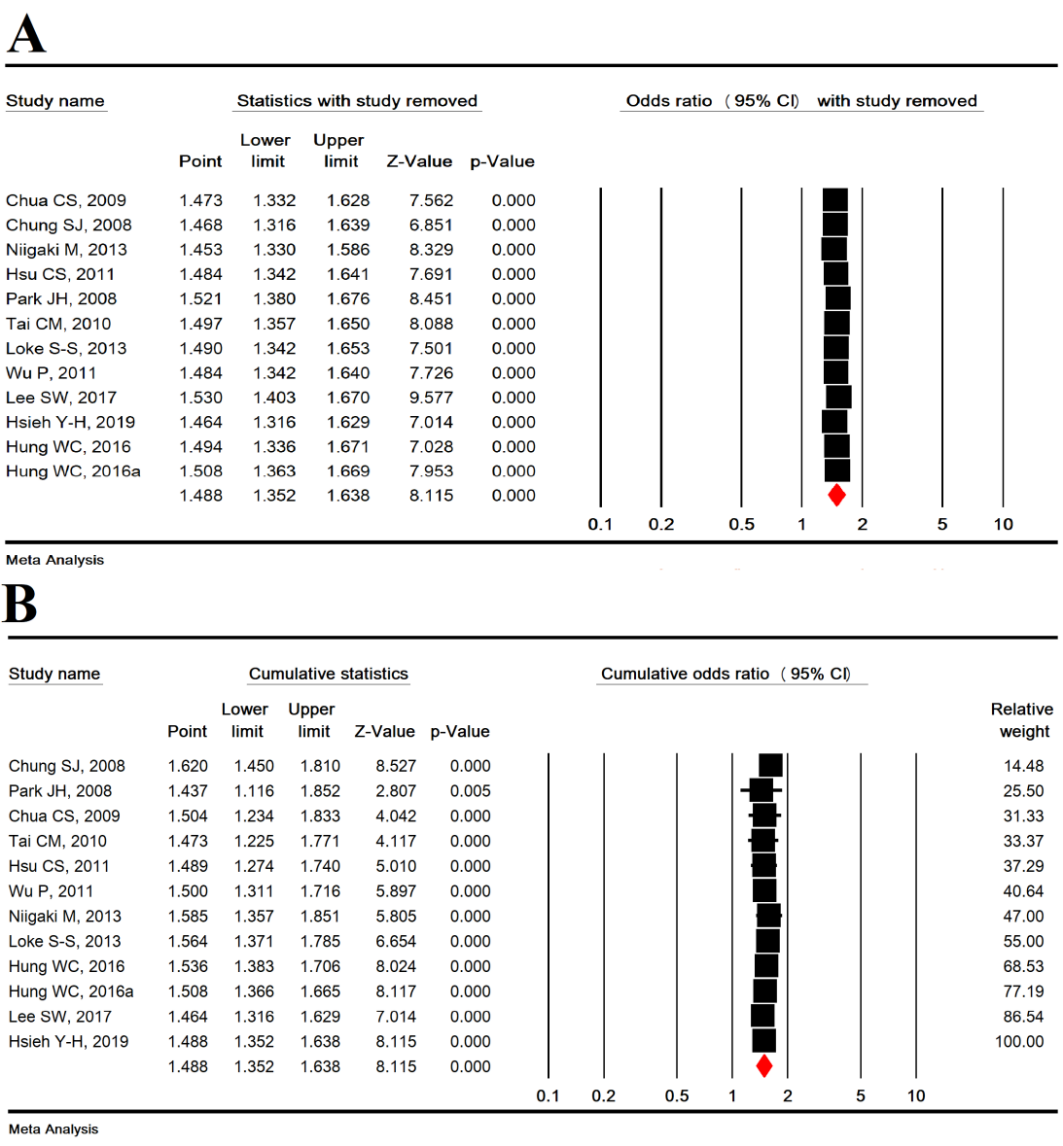
Sensitivity analysis (A) and cumulative analysis based on published year (B)

Sensitivity analysis was used to omit one study to show the overall estimation power and showed that the overall estimation is still robust (Figure 3A(Fig. 3); References in Figure 3: Chua et al., 2009[9]; Chung et al., 2008[10]; Hsieh et al., 2019[25]; Hsu et al., 2011[26]; Hung et al., 2016[27]; Lee et al., 2017[33]; Loke et al., 2013[34]; Niigaki et al., 2013[39]; Park et al., 2008[41]; Tai et al., 2010[48]; Wu et al., 2011[53]) and cumulative analysis based on the year of publication of the articles is shown in Figure 3B(Fig. 3).

### Subgroup analysis based on study type

**Figure 4:**
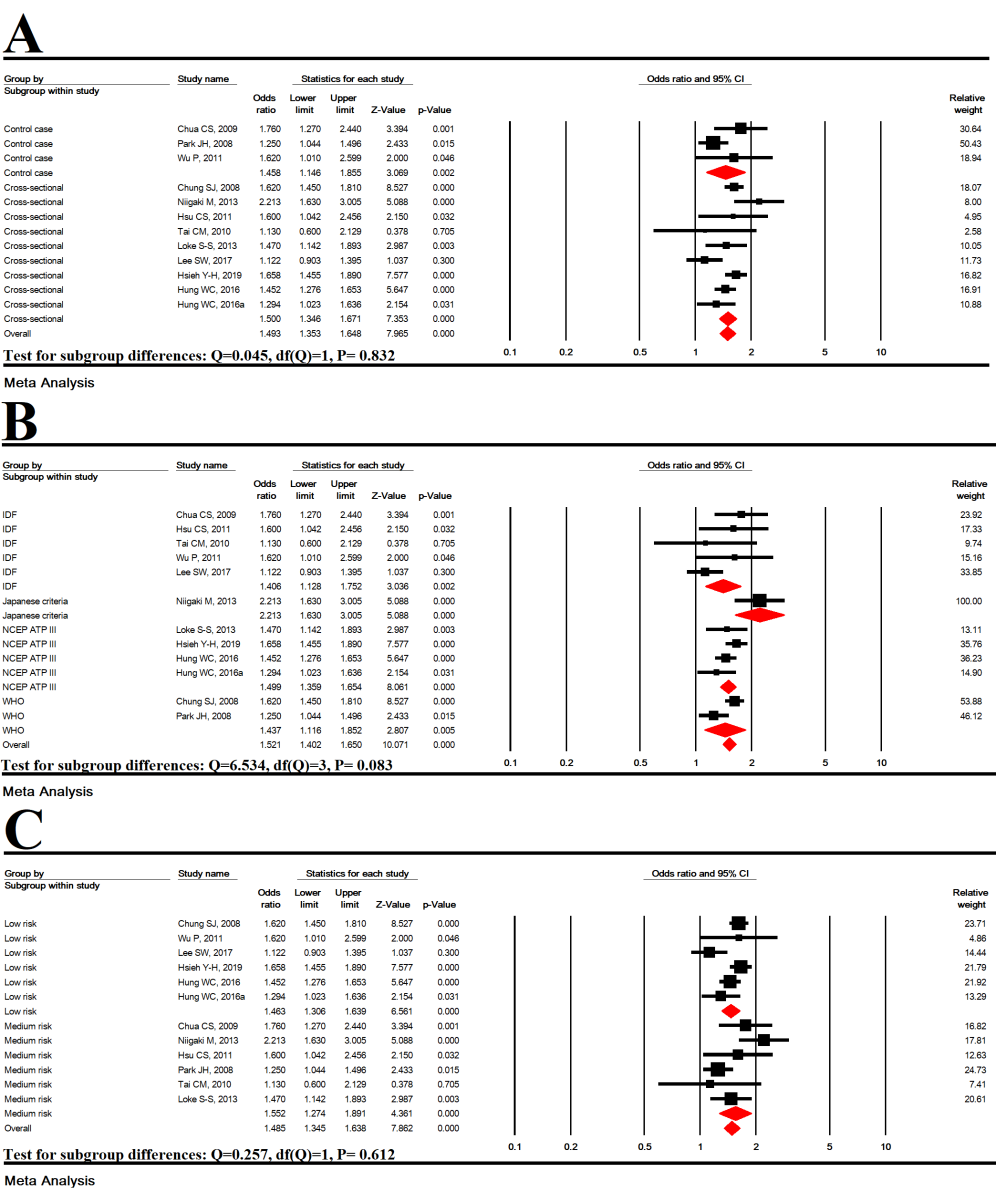
Subgroup analysis based on study type (A), MetS diagnostic criteria (B) and studies quality (C)

In cross-sectional (OR=1.458 [95 % CI: 1.146-1.855], P=0.002) and case-control (OR =1.500 [95 % CI: 1.346-1.671], P<0.001) studies, MetS was significantly associated with an increased risk of EE, but no significant difference was found between the types of studies (P = 0.832) (Figure 4A(Fig. 4); References in Figure 4: Chua et al., 2009[9]; Chung et al., 2008[10]; Hsieh et al., 2019[25]; Hsu et al., 2011[26]; Hung et al., 2016[27]; Lee et al., 2017[33]; Loke et al., 2013[34]; Niigaki et al., 2013[39]; Park et al., 2008[41]; Tai et al., 2010[48]; Wu et al., 2011[53]).

### Subgroup analysis based on MetS diagnostic criteria

According to MetS diagnostic criteria including IDF (OR=1.406 [95 % CI: 1.128-1.752], P=0.002), Japanese criteria (OR=2.213 [95 % CI: 1.630-3.005], P< 0.001), NCEP ATP III (OR=1.499 [95 % CI: 1.359-1.654], P<0.001), and WHO (OR=1.437 [95 % CI: 1.116-1.852], P= 0.005), MetS was significantly associated with an increased risk of EE, but no significant difference was found between the MetS diagnostic criteria (P=0.083) (Figure 4B(Fig. 4)).

### Subgroup analysis based on studies quality 

In studies with moderate quality (OR = 1.406 [95 % CI: 1.128-1.752], P<0.001) and high quality (OR=1.406 [95 % CI: 1.128-1.752], P<0.001), MetS was significantly associated with an increased risk of EE, but no significant difference was found (P=0.612) (Figure 4C(Fig. 4)).

### Meta-regression and publication bias

**Figure 5:**
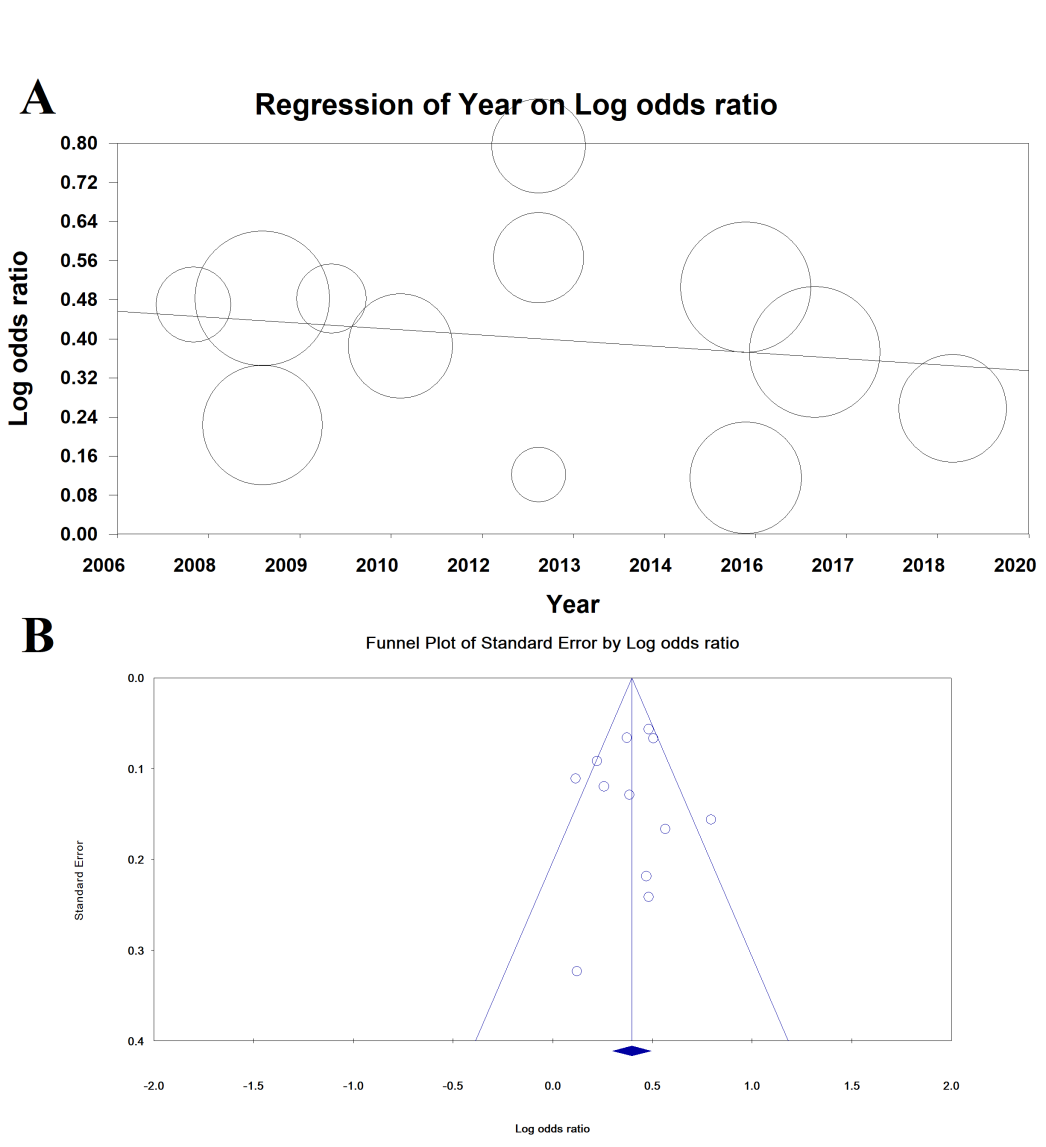
Meta-regression based on published year (A) and publication bias (B)

Meta-regression for the association between MetS and EE based on year of publication was not significant (meta-regression coefficient: -0.009 [95 % CI: -0.037 to 0.019], P = 0.529) (Figure 5(Fig. 5)).

Publication bias is shown as a funnel diagram in Figure 5(Fig. 5), and the Begg’s test (P= 0.945) and Egger's test (P=0.753) were not significant; therefore, publication bias did not play a role in the results.

## Discussion

The present study is the first meta-analysis that combined 12 primary studies to show that MetS was significantly associated with increased risk of EE. Subgroup analysis based on study design, MetS criteria and quality of studies was used to find the potential sources of heterogeneity, which showed no significant effect of the mentioned variables. In the included studies, the studies of Lee et al. in 2017[33] and Tai et al. in 2010[48] were not significant but the rest of the studies were significant (albeit with different significance levels) (Chua et al., 2009[9]; Chung et al., 2008[10]; Hsu et al., 2011[26]; Niigaki et al., 2013[39]; Park et al., 2008[41]; Hsieh et al., 2019[25]; Hung et al., 2016[27]). Moreover, a meta-analysis about the relationship between MetS and BE showed that MetS significantly increased the risk of BE, and suggested that future studies should focus on the treatment of metabolic syndrome based on the potential risk of BE and EA (He et al., 2016[21]).

Many other studies have reported a significant association between EE and risk factors, including MetS components, male gender, BMI=25, smoking, alcohol consumption, fasting blood sugar levels=126 mg/dl, and hiatal hernia (Cai et al., 2012[6]; Chung et al., 2008[10]; Kim et al., 2011[31]; Labenz et al., 2004[32]; Loke et al., 2013[34]; Wu et al., 2011[53]). However, the use of proton pump inhibitors (PPI) significantly reduces the risk of EE (Hosseinzadeh et al., 2011[23]).

Although the precise mechanism of EE in patients with MetS is not yet known (Chung et al., 2008[10]; Niigaki et al., 2013[39]), several mechanisms have demonstrated the association between each MetS component and the prevalence of EE. Studies have suggested that increased waist circumference (one of the most important components of MetS), as central, abdominal or visceral obesity, can independently increase the risk of EE (Chua et al., 2009[9]; Chung et al., 2008[10]; Hsu et al., 2011[26]; Loke et al., 2013[34]; Niigaki et al., 2013[39]; Tai et al., 2010[48]). Abdominal obesity causes metabolic disorders and can also contribute to EE development (Hung et al., 2016[27]; Souod et al., 2013[46]). Moreover, a meta-analysis confirmed that central obesity can be strongly associated with esophageal inflammation and reflux (Singh et al., 2013[45]). Studies have shown that visceral obesity can increase lower esophageal sphincter relaxation, the incidence of hiatal hernia, or even intra-abdominal pressure and acid reflux (Eross et al., 2018[15]; Wu et al., 2011[53]).

Hypertriglyceridemia has been associated with an increased risk of EE even after adjustments for obesity and other metabolic factors (Chua et al., 2009[9]; Park et al., 2008[41]). Impairment of lipid metabolism usually occurs in MetS. MetS is the result of obesity-related hormonal and systemic inflammatory changes and is associated with multiple systemic cancers in humans. There are several possible explanations for this relationship. First, fatty liver and insulin resistance may be responsible for hypertriglyceridemia, since liver fat has a significant association with fasting glucose and triglyceride levels (Nguyen-Duy et al., 2003[38]). Hypertriglyceridemia is also associated with increased insulin resistance (Hsu et al., 2011[26]; Ranjbar et al., 2016[43]). Second, since *Helicobacter pylori* infection is known to be a protective factor for EE disease (Cai et al., 2012[6]; Hosseinzadeh et al., 2011[23]; Hung et al., 2016[27]; Tsukada et al., 2006[50]) and chronic *H. pylori* infection can change serum lipid profile, including increased total cholesterol and triglycerides (Gudlaugsdottir et al., 2002[19]; Hunt et al., 2007[28]). Elevated serum TG levels can only be a side effect associated with H. pylori infection. 

In other studies, the association between hypertension and dyslipidemia (as MetS component) has also been demonstrated (Furuta et al., 2007[17]). In previous studies, atherosclerosis was associated with a high incidence of hiatal hernia. One of the causes of increased incidence of hiatal hernia is loss of flexibility of phrenoesophageal ligament in MetS patients. 

The present study has some limitations deserving acknowledgment. One of the limitations of the present study is the high heterogeneity of the studies, though we attempted to discover the cause of heterogeneity through subgroup analysis. Furthermore, most studies were conducted in Asian countries, which may influence the generalizability of the results. Finally the observational and prospective nature of the included studies precludes conclusion as to the causal nature of the observed association.

## Conclusions

MetS increases the risk of EE compared to control groups. Future studies should examine if MetS treatment reduces the risk of EE.

## Notes

Reza Ranjbar and Amirhossein Sahebkar (Biotechnology Research Center, Pharmaceutical Technology Institute, Mashhad University of Medical Sciences, Mashhad, Iran; E-mail: Amir_saheb2000@yahoo.com) contributed equally as corresponding author.

## Compliance with Ethical Standards

### Ethical Statement 

Not applicable.

### Conflict of interest

There is no conflict of interest between the authors.

### Acknowledgment

The authors would like to thank the Clinical Research Development Unit of Baqiyatallah Hospital, Tehran, Iran, for guidance and advice. We also would like to thank Ilam University of Medical Sciences for their financial support.

### Funding

None.

## Figures and Tables

**Table 1:**
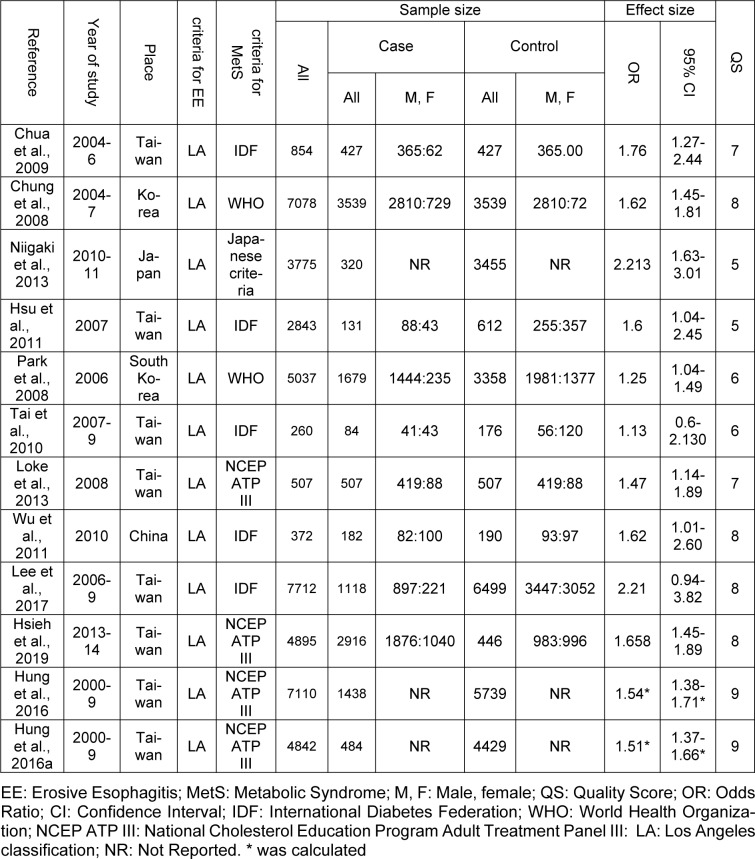
Summary of characteristics in studies into a meta-analysis
